# Computed Tomography Coronary Angiography as a Gatekeeper for Invasive Coronary Assessment Before Transcatheter Aortic Valve Implantation

**DOI:** 10.3390/medicina62040673

**Published:** 2026-04-01

**Authors:** Anastasios Apostolos, Nikolaos Ktenopoulos, Theoni Theodoropoulou, Panayotis Vlachakis, Paschalis Karakasis, Nikias Milaras, Panagiotis Iliakis, Andreas Synetos, George Latsios, Maria Drakopoulou, Grigorios Chrysostomidis, Grigorios Tsigkas, Konstantinos Toutouzas, Konstantinos Tsioufis, Vasileios Panoulas

**Affiliations:** 1 Department of Cardiology, Harefield Hospital, Royal Brompton and Harefield Hospitals, Guy’s and St Thomas’ NHS Foundation Trust, London UB9 6JH, UK; vasileios.panoulas2@nhs.net; 2 First Department of Cardiology, “Hippokration” General Hospital, National and Kapodistrian University of Athens, 11527 Athens, Greece; nikosktenop@gmail.com (N.K.); theoni.theodoropoulou98@gmail.com (T.T.); vlachakispanag@gmail.com (P.V.); panayiotisiliakis@gmail.com (P.I.); synetos@yahoo.com (A.S.); glatsios@gmail.com (G.L.); mdrakopoulou@hotmail.com (M.D.); ktoutouz@gmail.com (K.T.); ktsioufis@gmail.com (K.T.); 3 Second Department of Cardiology, “Hippokration” General Hospital, Aristotle University of Thessaloniki, 54642 Thessaloniki, Greece; pakar15@hotmail.com; 4 Department of Cardiology, “Hippokration” General Hospital, 11527 Athens, Greece; nikiasmilaras@gmail.com; 5 Department of Medicine, Division of Cardiology, Angiology and Internal Emergency Medicine, Knappschaft Kliniken University Hospital Bochum, Ruhr University Bochum, 44892 Bochum, Germany; 6 Faculty of Medicine, European University of Cyprus, Egkomi 2404, Cyprus; 7 Department of Cardiac Surgery, Onassis Cardiac Surgery Center, 17674 Athens, Greece; gregory.chrisostomidis@gmail.com; 8 Department of Cardiology, University Hospital of Patras, 26504 Patras, Greece; gregtsig@upatras.gr

**Keywords:** transcatheter aortic valve replacement, computed tomography coronary angiography, coronary artery disease, invasive coronary angiography

## Abstract

Transcatheter aortic valve implantation (TAVI) has become the predominant treatment strategy for severe aortic stenosis across all surgical risk categories. The coexistence of coronary artery disease (CAD) in 40–75% of TAVI candidates has traditionally mandated pre-procedural invasive coronary angiography (ICA). However, computed tomography coronary angiography (CTCA), which is already integral to TAVI planning for annular sizing and access route evaluation, offers the potential to assess coronary anatomy simultaneously. Accumulating evidence demonstrates that CTCA possesses excellent sensitivity (90–97%) and high negative predictive value (94–99%) for excluding significant proximal CAD, potentially serving as a reliable gatekeeper to avoid unnecessary ICA in a substantial proportion of patients. This approach is particularly attractive given the questionable benefit of routine pre-emptive coronary revascularization in stable TAVI candidates, as demonstrated by the ACTIVATION and NOTION-3 trials. This review synthesizes the current evidence on the diagnostic performance of CTCA, clinical outcomes with CT-guided strategies, technical considerations and limitations, and the evolving paradigm of coronary assessment in the contemporary TAVI era. We propose a practical algorithm integrating CTCA as a first-line screening tool, reserving ICA for patients with suspected significant proximal disease, thereby optimizing resource utilization while maintaining patient safety.

## 1. Introduction

Transcatheter aortic valve implantation (TAVI) has revolutionized the management of severe aortic stenosis (AS), evolving from a treatment reserved for inoperable patients to the preferred approach across all surgical risk categories [1,2,3,4,5]. The latest European Society of Cardiology (ESC) guidelines recommend TAVI as the first-line therapy for patients over 70 years unless anatomic contraindications exist for the transfemoral access [6]. This expansion of indications, as well as the aging population, has resulted in exponential growth in TAVI procedures globally, with volumes now exceeding surgical aortic valve replacement (SAVR) in many countries, including the United States [7].

Coronary artery disease (CAD) and degenerative AS frequently coexist, sharing common risk factors including advanced age, hypertension, diabetes mellitus, and dyslipidemia [8,9]. Contemporary registries report CAD prevalence ranging from 40% to 75% among TAVI candidates, establishing it as the most common cardiovascular comorbidity in this population [10,11]. The severity of CAD has been associated with worse mid- and long-term outcomes following TAVI, and intraprocedural hemodynamic instability during valve deployment might provoke ischemia in patients with significant coronary stenoses [12,13,14,15]. These considerations have traditionally mandated comprehensive coronary assessment prior to TAVI.

Invasive coronary angiography (ICA) has remained the cornerstone for CAD assessment, including pre-TAVI evaluation [16]. However, this approach carries several procedural risks, including vascular complications, contrast-induced nephropathy, and rare but serious events such as stroke and coronary dissection [17]. These risks are higher in the typical TAVI population, which consists of elderly, frail patients with multiple comorbidities and often impaired renal function. Furthermore, ICA adds logistical burden, prolongs hospital stay, and increases overall procedural costs [18].

Cardiac computed tomography (CT) has become indispensable in TAVI planning, providing a comprehensive assessment of aortic annular dimensions, root anatomy, valve calcification patterns, coronary ostial heights, and peripheral vascular access routes [19,20]. The natural extension of this examination is to include computed tomography coronary angiography (CTCA), which offers an attractive “one-stop-shop” approach that could potentially obviate the need for ICA in selected patients [21]. This review examines the current evidence supporting CTCA as a gatekeeper for invasive coronary assessment, addressing diagnostic performance, clinical outcomes, technical considerations, and practical implementation strategies.

## 2. Methods

This narrative review was based on a comprehensive literature search conducted in PubMed, MEDLINE, Embase, and the Cochrane Library from inception through January 2026. Search terms included combinations of “computed tomography coronary angiography,” “CTCA,” “CCTA,” “transcatheter aortic valve implantation,” “TAVI,” “TAVR,” “coronary artery disease,” and “invasive coronary angiography.” Additional references were identified from citation lists of key articles and recent meta-analyses. Studies evaluating the diagnostic accuracy of CTCA vs. ICA in TAVI candidates, clinical outcomes comparing CTCA-guided vs. ICA-based strategies, and relevant randomized trials were prioritized. This is a narrative review and was not conducted according to PRISMA guidelines.

## 3. The Role of CT in Pre-TAVI Evaluation

Electrocardiogram-gated CT with contrast enhancement represents the cornerstone of contemporary pre-TAVI assessment [22,23,24,25]. The examination provides critical information that directly affects procedural planning and device selection: accurate annular measurements for prosthesis sizing, assessment of aortic root geometry and calcification distribution, evaluation of coronary ostial heights to predict risk of coronary obstruction, characterization of left ventricular outflow tract calcification, and comprehensive iliofemoral vascular assessment for access route determination [19,20,26].

The integration of coronary artery assessment into the standard TAVI-CT protocol requires minimal modification to acquisition parameters. Modern multi-detector CT (MDCT) scanners, particularly those with 64 or more detector rows, provide sufficient spatial and temporal resolution for coronary evaluation [27]. The contrast-enhanced acquisition already performed for aortic root assessment typically achieves adequate coronary opacification, though optimization of bolus timing may enhance coronary visualization. Importantly, coronary assessment can be performed without additional contrast administration or radiation exposure, except that required for standard pre-TAVI CT [28].

The concept of CTCA as a gatekeeper relies on its ability to reliably exclude significant CAD, thereby identifying patients who can safely proceed to TAVI without ICA. This strategy leverages the high sensitivity and negative predictive value (NPV) of CTC, specifically its ability to accurately identify disease-free coronary segments. When CTCA demonstrates no significant proximal stenosis, additional invasive assessment might be required. Conversely, when CTCA identifies suspicious lesions or provides non-diagnostic images, ICA remains indicated for definitive evaluation.

## 4. Diagnostic Performance of CTCA for CAD Detection in TAVI Candidates

Multiple single-center studies have evaluated the diagnostic accuracy of CTCA for CAD detection in TAVI populations, consistently demonstrating favorable performance characteristics (Table 1). The study by Pontone and colleagues in 2011, examining 60 patients, reported a sensitivity of 88% and a specificity of 88% with an NPV of 91% [29]. Andreini et al. subsequently published a larger cohort of 325 patients, demonstrating improved diagnostic ability with a sensitivity of 90%, a specificity of 91%, and an NPV of 95% [30]. Hamdan et al. evaluated 115 patients and reported a sensitivity of 96% with an NPV of 96% [31], while Opolski and colleagues, in one of the largest single-center series (*n* = 475), achieved a sensitivity of 98% with an NPV of 94% [32]. Harris et al. similarly demonstrated a sensitivity of 99% and an NPV of 94% in their 100-patient cohort [33]. These studies consistently highlight a pattern: CTCA achieves excellent sensitivity for detecting significant CAD, with NPVs uniformly exceeding 90%, making it highly reliable for excluding disease.

Van den Boogert and colleagues conducted a systematic review and meta-analysis incorporating seven studies with a cumulative sample of 1275 patients [18]. The pooled patient-based analysis revealed a sensitivity of 95.3% (95% CI: 93.3–96.9%), a specificity of 65.3% (95% CI: 61.6–68.9%), a positive predictive value (PPV) of 70.8% (95% CI: 68.6–72.9%), and an NPV of 94.0% (95% CI: 91.6–95.8%). Critically, only 2.8% of patients had false-negative findings on CTCA. Using the routinely performed preoperative CT scans as a gatekeeper for coronary angiography could decrease additional coronary angiographies by 37% in this high-risk and fragile population.

The largest and most comprehensive evaluation comes from the Cleveland Clinic, where Kondoleon et al. analyzed 2217 patients undergoing both pre-TAVI CTA and ICA between 2015 and 2021 [18]. This study focused specifically on proximal coronary segments, considering that the anatomical territories are most amenable to revascularization and most relevant for clinical decision-making. At the ≥50% stenosis threshold, CTA demonstrated a sensitivity of 90%, a specificity of 92%, a PPV of 74%, and an NPV of 97%. Using the more clinically relevant ≥70% stenosis cutoff, performance improved further: a sensitivity 91%, a specificity 97%, a PPV 83%, and an NPV 99%. For bypass graft patency assessment, the sensitivity was 86%, the specificity was 97%, and the NPV was 98%. The Cleveland Clinic investigators calculated that CTA, as an effective screening tool, could have spared 51.8% of patients from invasive testing prior to TAVI. The Cohen Kappa analysis indicated substantial to near-perfect agreement between pre-TAVI CTA and ICA, validating the clinical utility of the non-invasive approach.

Notably, the consistently moderate specificity of CTCA in TAVI populations (ranging from 37% to 92% across studies) warrants further discussion. The high false-positive rate, driven predominantly by coronary calcification-related blooming artifacts, means that a proportion of patients will be unnecessarily referred for ICA despite having no hemodynamically significant disease. However, this trade-off must be contextualized within the intended clinical role of CTCA: its primary purpose is not to detect all CAD but rather to reliably exclude significant proximal disease, thereby identifying the subgroup that can safely proceed to TAVI without further invasive workup. The paradigm shifts from “detect all CAD” to “exclude proximal prognostically relevant CAD.” Accepting that some patients with false-positive findings will undergo confirmatory ICA is a tolerable downstream consequence given the much larger proportion safely spared from an invasive procedure altogether.

Furthermore, it is important to distinguish between the two stenosis thresholds used in the literature, as they serve different clinical purposes. The ≥50% threshold represents the conventional anatomical definition of “significant” CAD and is used in most diagnostic accuracy studies as the screening cutoff; its primary role is to identify lesions warranting further evaluation. On the other side, the ≥70% threshold (or FFR ≤ 0.80) represents the revascularization threshold, identifying lesions of sufficient severity to be considered for intervention. In the context of the CTCA-gatekeeper paradigm, the ≥50% threshold is applied as the screening cutoff: patients with no proximal stenosis ≥50% can safely proceed to TAVI, while those meeting or exceeding this threshold are referred for ICA, where the decision to revascularize is then based on the ≥70%/FFR criterion and individualized clinical assessment.

**Table 1:** Diagnostic performance of CTCA for CAD detection in TAVI candidates.

Study (Year)	*n*	Scanner Type	Sen (%)	Spe (%)	PPV (%)	NPV (%)	Acc (%)	Threshold
Pontone [29] (2011)	60	64-slice MDCT	88	88	85	91	88	≥50%
Andreini [30] (2014)	325	64-slice MDCT	90	91	81	95	91	≥50%
Hamdan [31] (2015)	115	256-slice MDCT (Brilliance iCT)	96	73	72	96	83	≥50%
Opolski [32] (2015)	475	2 × 64-slice DSCT (Definition)	98	37	67	94	—	≥50%
Harris [33] (2015)	100	2 × 128-slice DSCT (Definition Flash)	99	56	86	94	—	≥50%
Matsumoto [34] (2017)	60	320-row MDCT (Aquilion ONE)	92	58	60	91	72	≥50%
Gohmann [28] (2020)	388	2 × 192-slice DSCT (SOMATOM Force)	98	45	50	97	64	≥50%
van den Boogert [18] (2018) *	1275	Multiple (≥64-slice)	95	65	71	94	—	≥50%
Kondoleon [35] (2023)	2217	256-slice/DSCT (iCT, Force)	90	92	74	97	—	≥50%
Kondoleon [35] (2023)	2217	256-slice/DSCT (iCT, Force)	91	97	83	99	—	≥70%

* Meta-analysis data. Abbreviations: Acc, accuracy; CAD, coronary artery disease; CTCA, computed tomography coronary angiography; NPV, negative predictive value; PPV, positive predictive value; Sen, sensitivity; and Spe, specificity.

## 5. Clinical Outcomes with CTCA-Guided Strategies

The critical question extends beyond diagnostic accuracy to clinical outcomes: is it safe to proceed to TAVI without ICA when CTCA excludes significant proximal CAD? Chieffo et al. provided early evidence addressing this question in a landmark study from San Raffaele Hospital, Milan [36]. Among 491 TAVI patients, 375 (76.3%) underwent CTCA alone for CAD screening, while 116 (23.7%) required additional ICA due to suspicious or non-evaluable CT findings [34]. At 30 days and 1 year, there were no significant differences in major adverse cardiovascular and cerebrovascular events (MACCEs) between groups [34]. After multivariable adjustment for baseline and procedural confounders, CTCA alone was not associated with increased risk of a MACCE at 1 year (HR 0.89; 95% CI: 0.49–1.60; *p* = 0.69) [34]. Importantly, the periprocedural myocardial infarction rate was only 1.2%, which is comparable to rates reported in major TAVI trials where routine ICA was standard practice [3,36]. Additionally, patients screened with CTCA alone experienced shorter overall hospital stays (9 days vs. 10 days; *p* = 0.04), suggesting potential resource utilization benefits (Table 2).

The most recent evidence comes from Phichaphop et al., who evaluated 1165 patients undergoing TAVI at the Minneapolis Heart Institute [38]. The patients were categorized into TAVI-CTA (*n* = 464), where CTCA served as the gatekeeper, vs. conventional ICA (*n* = 701) groups [38]. By definition, all patients in the ICA group underwent invasive angiography, whereas only 47% of patients in the TAVI-CTA group proceeded to ICA based on CT findings, demonstrating that CTCA effectively served as a gatekeeper, sparing 53% of patients from invasive assessment. At the one-year follow-up, clinical outcomes were comparable between strategies. Symptom-driven revascularization occurred in 0.8% vs. 1.8% (*p* = 0.158) of patients, acute coronary syndrome in 1.6% vs. 1.7% (*p* = 0.846), and unplanned ICA in 2.7% vs. 2.8% (*p* = 0.767) for TAVI-CTA vs. ICA groups, respectively [38]. These findings provide robust contemporary evidence that a CT-first strategy maintains equivalent clinical safety while reducing procedural burden.

The safety of a CTCA-first strategy has now been validated at the meta-analytic level. Rahmati et al. recently published the first systematic review and meta-analysis comparing clinical outcomes of pre-TAVI CAD evaluation using CTCA vs. ICA [37]. Pooling data from five cohort studies encompassing 3073 patients (1573 CTCA-guided, 1500 ICA), the investigators found that pre-TAVI CTCA eliminated the need for ICA in 54.3% of patients. Importantly, no significant differences were observed between strategies at the 12-month follow-up for cardiac death (OR 0.89; 95% CI 0.45–1.73; *p* = 0.72), myocardial infarction (OR 0.83; 95% CI 0.36–1.96; *p* = 0.67), all-cause mortality (OR 0.86; 95% CI 0.60–1.24; *p* = 0.41), major adverse cardiovascular events (OR 0.85; 95% CI 0.50–1.45; *p* = 0.55), or unplanned revascularization (OR 0.50; 95% CI 0.22–1.15; *p* = 0.10). Notably, the CTCA-guided strategy was associated with significantly reduced rates of planned pre-TAVI PCI (OR 0.17; 95% CI 0.03–0.91; *p* = 0.03), reflecting the selective referral of only high-risk lesions for invasive assessment. Heterogeneity was negligible (I^2^ = 0%) for most outcomes, and leave-one-out sensitivity analysis confirmed the stability of effect estimates.

Beyond patient safety, the economic and logistical implications of a CTCA-gatekeeper strategy warrant further consideration. Avoiding ICA eliminates the need for additional catheterization laboratory time, reduces contrast exposure (minimizing nephrotoxicity risk in this vulnerable population), shortens referral-to-procedure intervals, and decreases overall procedural costs [39]. In healthcare systems facing capacity constraints, the ability to streamline pre-TAVI workup by eliminating unnecessary invasive procedures represents a significant advantage.

## 6. Technical Considerations and Limitations

The primary limitation of CTCA in TAVI populations is coronary artery calcification, which is ubiquitous in elderly patients with degenerative AS [40,41]. Calcified plaques create blooming artifacts that can overestimate stenosis severity, leading to false-positive findings and reduced specificity [42]. This phenomenon explains the consistently moderate specificity (60–70%) reported across studies despite excellent sensitivity. Several investigators have attempted to identify calcium burden thresholds that might predict non-diagnostic CTCA examinations. Rossi et al. suggested that diagnostic accuracy decreases at coronary artery calcium scores (CACSs) ≥400 [43], while Annoni and colleagues proposed this cutoff for deciding whether to attempt coronary assessment [44]. However, Gohmann et al. found extensive overlap between true-negative and false-positive results across calcium score ranges, concluding that no single threshold reliably predicts diagnostic utility [28]. The practical implication is that coronary calcification should be acknowledged as a limitation rather than an absolute contraindication to CTCA assessment (Table 3).

Optimal CTCA image quality requires low and regular heart rates, which are typically achieved with beta-blocker administration. However, patients with severe AS may not tolerate negative inotropic agents, and beta-blockers are often withheld due to concerns about hemodynamic compromise [35]. Similarly, sublingual nitroglycerin, which is routinely used to enhance coronary opacification through vasodilation, is contraindicated or requires caution in severe AS.

Atrial fibrillation (AF), which is present in approximately 30–40% of TAVI candidates, creates significant motion artifacts that degrade image quality. While modern reconstruction algorithms and wide-detector scanners with single-heartbeat acquisition have improved image quality in arrhythmic patients, AF remains associated with reduced diagnostic accuracy [45].

Temporal resolution is a critical determinant of CTCA image quality. Scanners with faster gantry rotation times (achieving temporal resolution < 300 ms) demonstrate superior diagnostic performance. Single-heartbeat CT systems significantly improved specificity compared with multi-heartbeat acquisition (82% vs. 60%; *p* < 0.0001), underscoring the importance of contemporary scanner technology [45]. Expert consensus documents now recommend high-temporal-resolution scanners for pre-TAVI coronary assessment [19].

Technical advances, including iterative reconstruction algorithms, deep learning image reconstruction, and motion correction technologies, continue to improve image quality. Seitz et al. evaluated high-pitch mode CTCA in 100 TAVI candidates, demonstrating diagnostic image quality in 30.3% of segments with a sensitivity of 75% and an NPV of 98.5%, highlighting both the potential and limitations of current technology in this challenging population [45].

An important practical consideration is that CTCA assessment in TAVI populations should focus on proximal coronary segments: the left main, proximal anterior descending, proximal left circumflex, and proximal right coronary arteries [18,46]. This approach aligns with current revascularization guidelines, which emphasize proximal disease as the primary determinant of prognostic benefit from intervention [6,47]. Mid and distal coronary segments are frequently non-diagnostic due to smaller vessel caliber and motion artifacts. Fortunately, disease in these locations is less clinically consequential.

Patients with prior coronary artery bypass grafting (CABG) represent a particularly important subgroup in the TAVI population, as they frequently present with incomplete documentation of graft anatomy and uncertain graft patency status. Paradoxically, CTCA demonstrates excellent diagnostic performance in this subgroup due to several favorable anatomical characteristics of bypass grafts: larger vessel diameter compared to native coronaries, relative immobility resulting in fewer motion artifacts, and sparse calcification, particularly in arterial conduits [48]. Modern CT technology demonstrates sensitivity and specificity values exceeding 95% for detecting graft stenoses, providing a non-invasive roadmap of graft number, location, and patency status. Two recent randomized controlled trials have examined the value of CTCA-guided invasive coronary angiography in patients with prior CABG. The BYPASS-CTCA trial was a single-center, open-label RCT conducted at Barts Heart Centre (United Kingdom) that randomized 688 patients with prior CABG requiring ICA to receive either CTCA before ICA or ICA alone [49]. CTCA-guided ICA resulted in a 56% relative reduction in procedural duration (17.4 vs. 39.5 min; *p* < 0.001), a significant reduction in contrast-induced nephropathy (3.2% vs. 8.8%; *p* = 0.003), and improved patient satisfaction scores. At the 3-year follow-up, patients in the CTCA-first arm demonstrated lower rates of major adverse cardiac events (35.8% vs. 43.5%; HR 0.73, 95% CI 0.58–0.93; *p* = 0.010) and reduced utilization of downstream imaging resources [49,50,51]. The GREECE trial was a multicenter RCT conducted across nine centers in Greece that randomized 251 patients with prior CABG (225 were included in the final analysis: 110 were CTCA-guided, and 115 were ICA-only) to evaluate the CTCA-directed strategy vs. the conventional ICA strategy [52]. Although total contrast volume was higher in the CTCA-guided group due to the additional CT acquisition (184.5 vs. 154 mL; *p* = 0.001), contrast administered during the invasive procedure itself was significantly reduced (101.5 vs. 154 mL; *p* < 0.001) [53]. Total fluoroscopy time was decreased with CTCA guidance (480 vs. 594 s; *p* = 0.027) [53]. The investigators concluded that CTCA-directed ICA expedites the invasive procedure and reduces fluoroscopy exposure, representing a valuable adjunctive strategy in this challenging population [53].

The Cleveland Clinic registry specifically evaluated bypass graft assessment in TAVI candidates, demonstrating an NPV of 98% for graft patency determination [18]. Hamdan et al. specifically analyzed the 23 patients (20%) with prior CABG in their cohort of 115 TAVI candidates [31]. The per-patient diagnostic yield in this subgroup was notably high, with a sensitivity of 100%, a specificity of 75%, a PPV of 95%, and an NPV of 100%. Andreini and colleagues similarly reported 100% accuracy of all bypass grafts (including seven left internal mammary arteries and nine saphenous vein grafts) in their 325-patient cohort [30]. These findings suggest that prior CABG should not be considered a contraindication to CTCA-based coronary screening; rather, post-CABG patients may represent an ideal population for this approach given the technical advantages of graft imaging, the added clinical value of comprehensive graft mapping prior to TAVI, and emerging RCT evidence supporting improved procedural efficiency with a CTCA-first strategy.

Patients with prior percutaneous coronary intervention (PCI) and coronary stents represent another important subgroup. Metallic stent struts create artifacts that can obscure the in-stent lumen, limiting assessment of in-stent restenosis. Stent evaluability is highly dependent on stent diameter (>3.0 mm generally assessable), material composition, and scanner resolution. In the Phichaphop et al. study, the presence of prior coronary stents was identified as a significant predictor of the need for subsequent ICA after CTCA [38]. Current evidence is insufficient to recommend CTCA as a reliable tool for in-stent assessment in TAVI candidates, and patients with prior PCI in proximal segments should generally be considered for ICA.

Several strategies have been proposed to mitigate the impact of coronary calcification. These include the use of high-definition CT acquisition modes, sharp reconstruction kernels, iterative metal artifact reduction algorithms, and subtraction techniques that digitally remove calcium from the coronary lumen. Dual-energy CT may offer additional benefit by improving tissue characterization and reducing blooming. In our proposed algorithm, heavily calcified segments that remain non-diagnostic despite optimized image reconstruction are classified as “non-evaluable” and trigger referral for ICA, thereby maintaining the safety-first philosophy of the gatekeeper approach.

Nevertheless, CTCA should not be considered a panacea. Several important limitations of the current evidence base warrant explicit acknowledgment. First, the vast majority of diagnostic accuracy studies are single-center, retrospective analyses with varying patient selection criteria, scanner technologies, and stenosis thresholds, limiting the generalizability of pooled performance estimates. Second, the outcome studies comparing CTCA-guided vs. ICA-based strategies are observational and subject to inherent selection bias: patients routed to ICA likely had higher-risk coronary anatomy or non-diagnostic CT findings, creating non-comparable groups. Third, no prospective randomized trial has yet directly compared a CT-first vs. an ICA-first pre-TAVI strategy with clinical outcomes as the primary endpoint. These limitations underscore the need for well-designed, multicenter RCTs to provide definitive evidence.

## 7. The Revascularization Question: Rethinking the Need to Detect CAD

The rationale for CAD assessment before TAVI has traditionally been predicated on the assumption that identifying and treating significant stenoses improves outcomes. However, recent evidence has substantially challenged this premise, suggesting that routine pre-emptive revascularization of stable CAD confers limited benefit in TAVI recipients [54,55,56,57,58,59]. More specifically, the ACTIVATION trial randomized 235 patients with significant CAD (≥70% stenosis in a major epicardial vessel) to percutaneous coronary intervention (PCI) before TAVI vs. no PCI prior to TAVI [60]. The primary composite endpoint of all-cause death or rehospitalization at 1 year occurred in 41.5% of the PCI arm vs. 44.0% of the no-PCI arm [60]. The requirement for non-inferiority was not met (difference: −2.5%; one-sided upper 95% confidence limit: 8.5%; *p* = 0.067). It is noteworthy that mortality was similar between groups (13.4% vs. 12.1%), and PCI was associated with significantly higher rates of any bleeding (*p* = 0.021) [60]. Important limitations of ACTIVATION include prolonged enrollment (2012–2017), exclusion of patients with left main disease and more severe angina (CCS Class > 2), and the absence of physiology assessment. Nevertheless, this trial was the first to provide randomized evidence regarding the benefit of routine pre-TAVI revascularization.

Recently, the NOTION-3 trial enrolled 455 TAVI candidates with significant CAD (defined as FFR ≤ 0.80 or visual stenosis ≥ 90%) between 2017 and 2022. Patients were randomized to FFR-guided complete revascularization plus TAVI vs. TAVI alone [61]. At a median follow-up of 2 years, the primary composite endpoint of death, myocardial infarction, or urgent revascularization occurred in 26% of the PCI group vs. 36% of the conservative group (HR 0.71; 95% CI: 0.51–0.99; *p* = 0.04). While the primary endpoint favored PCI, this was driven primarily by the reduction in urgent revascularization (2% vs. 11%; HR 0.20; *p* < 0.001) and myocardial infarction (8% vs. 14%; HR 0.54; *p* = 0.037) [61]. It is noteworthy that there was no difference in all-cause mortality between groups. Furthermore, bleeding events were significantly higher in the PCI group (28% vs. 20%; HR 1.51; *p* = 0.035) [61]. The NOTION-3 investigators concluded that while PCI provides benefit in selected patients with physiologically significant CAD, the decision should be individualized based on age, comorbidities, bleeding risk, and lesion complexity, and after discussion with the Heart Team [61].

The findings from ACTIVATION and NOTION-3 have profound implications for pre-TAVI coronary assessment strategies. These trials demonstrate that routine revascularization of stable CAD provides modest clinical benefit at the cost of increased bleeding, particularly when PCI is performed empirically without physiological guidance [62]. This paradigm shift strongly supports the CTCA-gatekeeper approach. CTCA excels at excluding significant proximal disease, which is mainly the information needed for safe procedural planning. The moderate specificity and tendency to overestimate stenosis severity become less problematic when the clinical question shifts from “does this patient need revascularization?” to “can this patient safely proceed to TAVI without invasive assessment?” [63].

It is critical to recognize that the role of CTCA in the pre-TAVI setting is that of an anatomical screening tool for excluding significant proximal disease, rather than a modality for guiding lesion-level revascularization decisions. CTCA cannot reliably determine the hemodynamic significance of intermediate stenoses, and functional assessment (invasive FFR or emerging CT-FFR) remains necessary when revascularization is being considered.

## 8. Current Guidelines and Expert Consensus

The 2025 ESC/EACTS Guidelines for the Management of Valvular Heart Disease represent a paradigm shift in pre-procedural coronary assessment, formally endorsing a “CT-first approach” for CAD evaluation before valve interventions [6]. For patients with moderate or lower (≤50%) pretest probability of obstructive CAD, CTCA is now recommended before valve intervention (Class I, Level of Evidence B), which is a significant progress from prior guidance that mandated invasive coronary angiography for all patients over age 40. Specifically for TAVI candidates, the guidelines state that omission of invasive coronary angiography should be considered if procedural planning CT angiography is of sufficient quality to exclude relevant CAD (Class IIa, Level of Evidence B). The guidelines acknowledge that while CTCA sensitivity for detecting obstructive CAD is high (95–97%), specificity remains modest (68–73%), which is attributable to the high prevalence of coronary artery calcification and AF in patients with severe AS, factors that limit imaging resolution and interpretability. Invasive coronary angiogram remains recommended for patients with high or very high (>50%) pretest likelihood of obstructive CAD and for assessment in patients with severe ventricular secondary mitral regurgitation.

The 2025 guidelines also incorporate evidence from the NOTION-3 trial regarding concomitant CAD management, demonstrating that routine PCI in TAVI patients with ≥90% stenosis (or FFR ≤ 0.80) was associated with reduced risk of a composite endpoint including all-cause death, myocardial infarction, and urgent revascularization at the 2-year follow-up [6]. Notably, the age threshold for preferential TAVI over SAVR was lowered from 75 to 70 years, reflecting the accumulation of randomized evidence of mid-term safety and efficacy in lower-risk populations and further expanding the cohort of patients who will require pre-procedural CT angiography as the standard pathway [6]. These updated recommendations, alongside data supporting CTCA as an effective gatekeeper, position non-invasive imaging at the center of TAVI workup algorithms for the first time in major society guidelines [6].

## 9. Practical Algorithm: Implementing a CT-First Strategy

**Figure 1:**
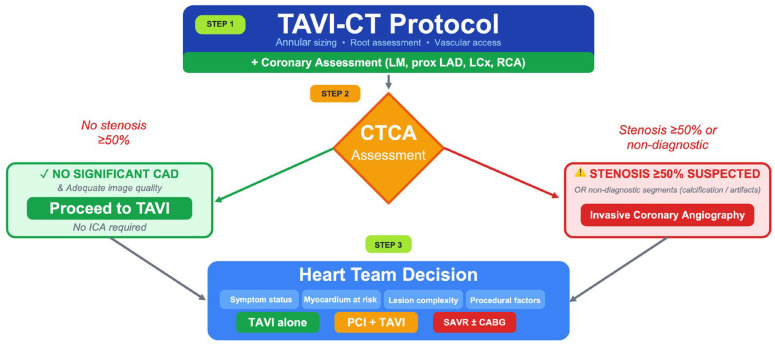
Proposed TAVI-CT protocol for coronary assessment in patients with suspected coronary artery disease. This algorithm outlines a streamlined TAVI-CT protocol for preoperative coronary assessment using low-dose coronary CT angiography (CTCA). Patients without significant coronary artery disease (CAD) on initial CTCA proceed directly to TAVI, whereas those with stenosis of 50% or greater, or suspected non-significant CAD, undergo invasive coronary angiography. Multidisciplinary heart team decisions then guide treatment: TAVI alone, TAVI with percutaneous coronary intervention (PCI), or TAVI with coronary artery bypass grafting (CABG).

Based on the accumulated evidence, we propose the following algorithm for coronary assessment in TAVI candidates (central illustration) (Figure 1):

**Step 1:** All patients undergo standard TAVI-CT with contrast enhancement for annular sizing, root assessment, and vascular access evaluation. Coronary artery assessment is performed simultaneously, focusing on the left main, proximal LAD, proximal LCx, and proximal RCA arteries.

**Step 2:** If CTCA demonstrates no significant stenosis (≥50%) in proximal segments and adequate image quality, the patient proceeds directly to TAVI without ICA.

**Step 3:** If CTCA identifies suspicious stenosis (≥50%) in any proximal segment, or if proximal segments are non-diagnostic due to calcification or artifacts, ICA is performed for definitive assessment.

**Step 4:** Decisions regarding revascularization are made by the Heart Team on an individualized basis, considering symptom status, myocardium at risk, lesion complexity, and procedural factors.

### Central Illustration

The proposed algorithm for coronary assessment in TAVI candidates using CTCA as a gatekeeper: All patients undergo standard TAVI-CT with coronary assessment. Patients with no significant proximal stenosis on CTCA proceed directly to TAVI. Patients with suspicious findings or non-diagnostic segments undergo ICA for definitive evaluation. The Heart Team makes individualized decisions regarding revascularization based on clinical context, symptoms, and anatomical findings.

## 10. Future Directions

Despite the great improvement in the diagnostic role of CTCA, further investigation is required. The EASE-IT CT registry represents an important ongoing effort to validate the CTCA-gatekeeper strategy in routine clinical practice [64]. This multicenter European registry is enrolling patients aged ≥75 years with severe AS scheduled for TAVI with SAPIEN family devices. The study will consist of two prospective cohorts: a CTA-only cohort, where significant left main/proximal LAD stenosis is ruled out on CT, and a CTA+ICA control cohort [64]. The registry will provide real-world data on the safety and efficacy of deferring ICA based on CTCA findings [64]. Furthermore, CT-derived fractional flow reserve (CT-FFR) offers the potential to provide both anatomical and functional coronary assessment non-invasively [65,66,67]. However, data regarding CT-FFR in severe AS patients remain extremely limited. The hemodynamic perturbations associated with AS, such as increased left ventricular end-diastolic pressure and altered coronary flow patterns, may affect the accuracy of CT-FFR calculations. Until validation studies are completed, CT-FFR cannot be routinely recommended in this population [68,69,70,71,72]. Additionally, artificial intelligence and machine learning applications are being developed to enhance CTCA interpretation, including automated stenosis quantification, calcium scoring, and plaque characterization [73,74,75]. Deep learning algorithms may help overcome some limitations related to image quality and calcification artifacts [76,77,78,79]. Recent studies have demonstrated improved diagnostic accuracy with deep learning image reconstruction and motion correction algorithms, suggesting a promising direction for future development. While observational data strongly support the safety of a CTCA-gatekeeper approach, randomized controlled trials comparing CT-first vs. ICA-first strategies would provide definitive evidence. The ongoing COMPLETE-TAVR trial (clinicaltrials.gov: NCT04634240), a 4000-patient study comparing staged complete revascularization vs. medical therapy after TAVI, will provide additional insights into the management of concomitant CAD.

## 11. Conclusions

In conclusion, CTCA may represent a safe, effective, and efficient approach to coronary assessment in TAVI candidates. By serving as a gatekeeper, CTCA has the potential to reduce unnecessary invasive procedures, minimize patient burden, and optimize healthcare resource utilization while maintaining clinical safety. However, it should be acknowledged that current evidence is predominantly derived from retrospective, single-center studies, and prospective randomized trials are needed to definitively establish the superiority or non-inferiority of a CT-first approach compared with routine ICA. Until such data are available, the adoption of CTCA-guided strategies should be individualized, guided by Heart Team discussion, and implemented in centers with appropriate CT expertise and scanner technology.

## Figures and Tables

**Table 2:** Clinical outcome studies comparing CTCA-based vs. ICA-based Pre-TAVI assessment.

Study	*n*	Design	Key Findings	Conclusions
Chieffo [36] (2015)	491	Retrospective single-center	MACCE at 1 y: CTCA-only 15.4% vs. CTCA + ICA 18.1% (*p* = NS); HR 0.89 (0.49–1.60)	CTCA is safe as the first-line screening test; shorter LOS in the CTCA-only group
Gohmann [28] (2020)	460	Retrospective single-center	AKI was lower in the CTCA-only group; no MI in the CTCA-only group	CTCA-based screening is safe and may reduce complications
Phichaphop [37] (2025)	1165	Retrospective single-center	ACS at 1 y: 1.6% vs. 1.7% (*p* = NS); Revasc: 0.8% vs. 1.8% (*p* = NS)	CTCA-gatekeeper strategy spared 53% from ICA with equivalent outcomes

Abbreviations: ACS, acute coronary syndrome; AKI, acute kidney injury; CI, confidence interval; CTCA, computed tomography coronary angiography; HR, hazard ratio; ICA, invasive coronary angiography; LOS, length of stay; MACCEs, major adverse cardiovascular and cerebrovascular events; MI, myocardial infarction; NS, not significant; and TAVI, transcatheter aortic valve implantation.

**Table 3:** Comparison of CTCA vs. ICA strategies before TAVI.

Domain	CTCA	ICA
Primary purpose	Exclude clinically relevant proximal obstructive CAD and triage those who need ICA	Universal invasive coronary assessment before TAVI
Diagnostic role	Best suited for rule-out (high sensitivity/very high NPV)	Gold standard anatomical assessment
Sensitivity	High (particularly for LM/proximal segments)	Very high
Specificity	Moderate (calcification-related false positives)	High
LM/proximal LAD assessment	Very good	Excellent
Mid/distal vessel assessment	Often limited by motion, small caliber, artifacts	Excellent
Impact of heavy calcification	Major limitation (blooming → overestimation)	Minimal impact
Impact of atrial fibrillation	Reduced image quality in many cases	No major impact
Need for heart rate control	Often desirable; beta-blockers may be limited in severe AS	Not required
Contrast burden (overall pathway)	Often lower (coronary assessment integrated into routine TAVI-CT)	Higher (ICA adds an additional contrast procedure)
Renal outcomes/AKI risk	Potentially lower AKI risk by avoiding unnecessary ICA	Higher AKI risk due to additional contrast exposure
Vascular access complications	None (non-invasive)	Present (particularly in elderly/vasculopathic patients)
Risk of stroke/coronary dissection	Extremely low	Low but non-zero
Workflow efficiency	“One-stop-shop”; may shorten pre-TAVI work-up	Requires cath-lab scheduling and additional procedures
Cost and resource utilization	Potential reduction in cath-lab use and costs	Higher cath-lab and staffing burden
Value for TAVI planning	Provides annulus/root/access + coronary heights simultaneously	Coronary anatomy only
Coronary obstruction risk prediction	Excellent (CT-based)	Limited
Future coronary access after TAVI	Highly informative (frame–ostia relationship, commissural alignment planning)	Not assessed
Best suited for	Stable patients, low–intermediate CAD likelihood, goal of excluding proximal disease	High pretest probability, unstable symptoms/ACS, non-diagnostic CT, complex clinical scenarios
Main limitation	Calcification, AF, motion artifacts → non-diagnostic scans or false positives	Invasiveness, contrast load, procedural complications

## Data Availability

No new data were created or analyzed in this study.

## Abbreviations

The following abbreviations are used in this manuscript:

AF	Atrial Fibrillation
AKI	Acute Kidney Injury
AS	Aortic Stenosis
CABG	Coronary Artery Bypass Grafting
CAD	Coronary Artery Disease
CACS	Coronary Artery Calcium Score
CT	Computed Tomography
CTCA	Computed Tomography Coronary Angiography
CT-FFR	CT-derived Fractional Flow Reserve
ESC	European Society of Cardiology
ICA	Invasive Coronary Angiography
LAD	Left Anterior Descending
LM	Left Main
MACEs	Major Adverse Cardiovascular Events
MACCEs	Major Adverse Cardiovascular and Cerebrovascular Events
MDCT	Multi-Detector Computed Tomography
NPV	Negative Predictive Value
PCI	Percutaneous Coronary Intervention
PPV	Positive Predictive Value
SAVR	Surgical Aortic Valve Replacement
TAVI	Transcatheter Aortic Valve Implantation
